# Targeting a Hidden Enemy: Pyriproxyfen Autodissemination Strategy for the Control of the Container Mosquito *Aedes albopictus* in Cryptic Habitats

**DOI:** 10.1371/journal.pntd.0005235

**Published:** 2016-12-29

**Authors:** Kshitij Chandel, Devi Shankar Suman, Yi Wang, Isik Unlu, Eric Williges, Gregory M. Williams, Randy Gaugler

**Affiliations:** 1 Center for Vector Biology, Rutgers University, 180 Jones Ave., New Brunswick, NJ, United States of America; 2 Mercer County Mosquito Control, West Trenton, NJ, United States of America; 3 Essex County Division of Environmental Affairs, Cedar Grove, NJ, United States of America; 4 Hudson Regional Health Commission, 595 County Ave., Secaucus, NJ, United States of America; Mahidol University, THAILAND

## Abstract

**Background:**

The Asian tiger mosquito, *Aedes albopictus*, is a vector of dengue, Chikungunya, and Zika viruses. This mosquito inhabits a wide range of artificial water-holding containers in urban and suburban areas making it difficult to control. We tested the hypothesis that female-driven autodissemination of an insect growth regulator could penetrate cryptic oviposition habitats difficult to treat with conventional insecticidal sprays.

**Methodology:**

Oviposition preferences of *Ae*. *albopictus* females for open and cryptic cups were tested in semi-field experiments. Two conventional larvicidal sprayers were tested to determine droplet penetration and larvicidal efficacy in open and cryptic habitats using *Bacillus thuringiensis* var. *israelensis* (*Bti*) in the field. Finally, the efficacy of pyriproxyfen autodissemination stations was assessed in cryptic and open cups in residential areas during 2013 and 2014.

**Principal Findings:**

Gravid females strongly preferred cryptic (53.1±12.9 eggs/cup) over open (10.3±4.3 eggs/cup) cups for oviposition. Cryptic cups showed limited droplet penetration and produced 0.1–0.3% larval mortality with a conventional backpack and low-volume sprays of *Bti*. The autodissemination stations effectively contaminated these cryptic cups (59.3–84.6%) and produced 29.7–40.8% pupal mortality during 2013–2014. Significant pupal mortality was also observed in open cups.

**Conclusions:**

The autodissemination station effectively exploits the oviposition behavior of wild gravid females to deliver pyriproxyfen to targeted oviposition habitats. Although the pupal mortality in cryptic cups was relatively lower than expected for the effective vector control. Autodissemination approach may be a suitable supporting tool to manage *Ae*. *albopictus* immatures in the cryptic habitats those are less accessible to conventional larvicidal sprays.

## Author Summary

*Aedes albopictus* is a highly invasive mosquito species and a competent vector of dengue, Chikungunya, Zika and other arboviruses. In the absence of therapeutics, vector control is the only means of controlling these diseases. Larvicides are important tool to limit mosquito populations. Backpack and low volume sprayers are heavily used for ground-based larvicide applications, but their potential to reach hidden or cryptic larval habitats is not well established. In the present study, we found that *Ae*. *albopictus* shows a strong oviposition preference for cryptic cups. Moreover, cryptic cups received limited droplet penetration using conventional spray methodologies, resulting in almost negligible larval mortality as compared to open cups in field experiments. We further evaluated the efficacy of an autodissemination station to deliver the insect growth regulator pyriproxyfen to cryptic cups in field experiments. Autodissemination shows good efficacy in transferring toxic concentrations of pyriproxyfen to cryptic cups and may provide a significant advantage over sprayers in delivering insecticide to the cryptic larval habitats strongly preferred by *Ae*. *albopictus*.

## Introduction

*Aedes albopictus* is a highly invasive mosquito species that has originated from South East Asia and now has spread in most part of the world[1]. In the United States, this mosquito species was first reported from Texas in 1985 and has since expanded its range to more than 30 states [2]. *Aedes albopictus* (Skuse) is a major public health problem due to its ability to transmit the dengue, chikungunya, yellow fever viruses, and is a competent vector for at least 22 other arboviruses including Zika [3]. There are more than 50 million dengue cases annually, and around 2.5 billion peoples are living at the risk of infection [4].

Source reduction, adulticides and larvicide applications are routinely used to manage Asian tiger mosquitoes [5]. Adulticides are very effective in suppressing adult populations but populations tend to quickly rebound [6,7]. Source reduction by eliminating potential larval habitats is also a key element of mosquito control. Eliminating the diverse array of containers used by peridomestic *Ae*. *albopictus*, including discarded tires, trash, soda cans, plant pots, bird baths, corrugated drain pipes etc., is extremely challenging and labor intensive [5,6]. In addition, mosquito control agencies find it difficult to get access to the private properties where these habitats are located [8]. Moreover, accumulation of containers such as beverage cans and other trash are hard to manage because they are often rapidly re-generated. Larvicide application is an effective alternative to control immature *Ae*. *albopictus* in larval habitats. Larvicides are applied with backpack or larger motorized sprayer such as a truck-mounted low and ultralow volume sprayers [9,10]. For instance, *Bacillus thuringiensis* var. *israelensis* is a well-established larvicide used for mosquito control, particularly in urban areas, with proven efficacy [8,10]. It is imperative for any larvicide application to reach concealed cryptic habitats, where this mosquito prefers to oviposit. However, the effectiveness of conventional larvicide equipment to deliver larvicide in such cryptic habitats is not well documented. There is no information available regarding the efficacy of conventional methods to deliver larvicides into cryptic larval habitats.

Autodissemination using the insect growth regulator pyriproxyfen, a juvenile hormone mimic, has recently emerged as a potential new vector management tool. This “pull-and-push” method exploits the skip oviposition behavior [11] of the Asian tiger mosquito to treat water-holding containers [12]. We have developed an autodissemination station that attracts gravid females and contaminates them topically with pyriproxyfen as they exit the station to locate new oviposition sites where the insecticide is then transferred [12].

We ask if *Ae*. *albopictus* prefers cryptic oviposition cups over the open cups, and that if cryptic oviposition cups are better targeted by autodissemination approach compared to conventional spray technologies. We examined *Ae*. *albopictus* oviposition preference for cryptic and open cups in a large cage field experiment; tested the efficacy of larvicidal sprayers to treat cryptic cups through droplet penetration and larvicide application, and then examined autodissemination efficacy in open versus cryptic cups against field populations of the Asian tiger mosquito.

## Methods

### Mosquito Culture

A laboratory colony of *Ae*. *albopictus* mosquito was maintained at the Center for Vector Biology, Rutgers University. Eggs were originally field collected from Mercer and Monmouth Counties, New Jersey in 2008. Mosquitoes were reared at 26 ± 1°C temperature and 75 ± 5% relative humidity under 16L:8D photoperiod. Brewer’s yeast was provided as a larval food (30 mg/L) daily. Adult mosquitoes were fed on 10% sucrose solution and guinea pigs were used for the blood feeding. Previously, a baseline data for the susceptibility of the laboratory-raised colony of *Ae*. *albopictus* to pyriproxyfen has been established [13].

### Ethics Statement

The study protocol was approved by the Institutional Animal Care and Use Committee (IACUC), Rutgers University. The study was performed in strict accordance with IACUC Animal Use Protocol # 86–129.

### Oviposition preference

**Fig 1:**
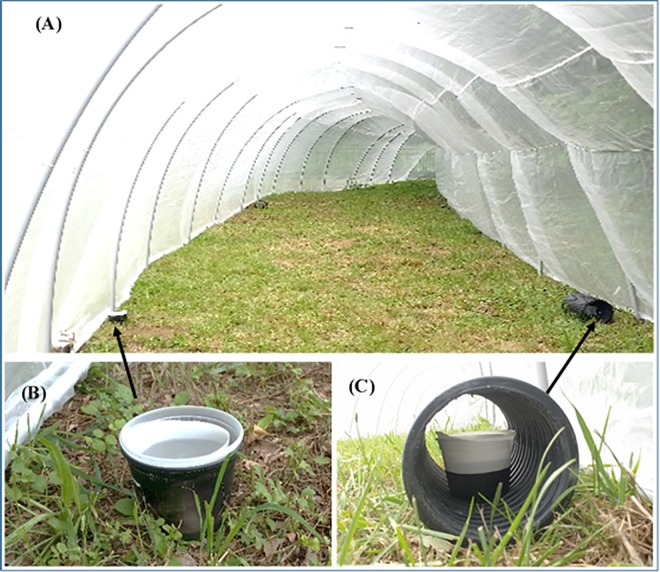
Large field cage (A) for assessing the oviposition preference of *Ae*. *albopictus* females in open (B) *vs*. cryptic (C) sentinel oviposition cups.

The oviposition preferences of gravid *Ae*. *albopictus* females were tested in a semi-field experiment conducted in a 50 (L) x 3 (W) x 2 (H) m mosquito proof tunnel cage (Fig 1A) in an open area surrounded by trees and bushes. Five plastic cups (450 ml capacity) were used as open oviposition cups (Fig 1B), whereas another five cups inserted into plastic pipes (45 (L) x 15 (diam) cm) were used as cryptic cups (Fig 1C). Each sentinel cup held 250 ml dechlorinated tap water and was lined with filter paper (Whatman filter paper #1) as an oviposition substrate. Open and cryptic cups were placed alternatively every 10 m distance at 15 cm away from the cage side walls (Fig 1A). Two hundred gravid females (4–5 days post-blood feeding) were released at the center of the cage. After 5 days, the cups were transported to the lab. Oviposition preference was determined by counting the number of cups that received eggs and the number of eggs that were accumulated in each cup. To nullify positional preference, open and cryptic cups were switched in each experiment. The experiment was repeated three times during June–July 2015.

### Droplet penetration

Two conventional larvicide applicators, a Stihl^®^ SR 420 backpack sprayer (Andreas Stihl Ag & Co. KG, Waiblingen, Germany) and a truck-mounted Curtis Dyna-Fog Ag-Mister LV- 8™ (Curtis Dyna-Fog, Westfield, IN, USA) low-volume (LV) sprayer were evaluated for their efficacy to deliver spray droplets in open and cryptic cups. Tests were carried out in an open parking lot at Rutgers University. Cryptic cups (i.e., ovicups inserted into pipes) were placed in parallel, perpendicular and oblique positions relative to the equipment’s spray direction. Cups were placed 1 m distance apart in three replicates. Kromekote papers (Mohawk Fine Papers, Inc., Cohoes, NY, USA) measuring 5×7.5 cm were placed in the bottom of each cup. A 2% solution of Red 40 granular dye (Glanbia Nutritionals, Carlsbad, CA, USA) was sprayed using the backpack and truck-mounted sprayers. Experiments were conducted on the day when the prevalent wind speed was minimal and was found to be between 2 to 5 km/h. Backpack sprayer was calibrated to flow rate of 0.12 L/min and application was performed from the distance of 3 m from the cup placement. The low-volume sprayer was adjusted to flow rate of 8.3 L/min and application was performed at 10 m from the cup placement. Both sprayers moved at the speed of 8 km/h and perpendicular to the wind direction. Papers were collected after 30 min of application and analyzed by DropVision AG system (Leading Edge Associates, Inc., Waynesville, NC) to determine droplet size and density. Each experiment was repeated three times.

### Sprayer field efficacy

Two field trials were carried out to evaluate *Bacillus thuringiensis* var. *israelensis* (*Bti*) (VectoBac^®^ WDG, Valent BioScience Corp., Libertyville, IL, USA) larvicide penetration in open and cryptic cups in Essex County (New Jersey, USA) in 2013 with a backpack sprayer and in Mercer County (New Jersey, USA) in 2014 with truck-mounted LV sprayer. Whereas the Essex trial was conducted in a suburban area, the Mercer trial was performed in an inner city urban area. The study areas were divided into one control and three treatment plots of ca. 4000 m^2^ per plot. Control plot was 1000 m apart from the treatment plots. Open and cryptic cups (12 cups per plot) were placed in shaded areas and filled with 250 ml of dechlorinated water. Larvicide applications were then made by the local county mosquito control agencies under the authority of Title 26.9 of the New Jersey administrative code. Larvicide was applied at the application dose of 1 kg/ha with flow rate of 0.12 L/min and 8.3 L/min for backpack and LV sprayer, respectively. Both the sprayers were moving at the speed of 8 km/h. The control plot received five open cups.

Cups were collected 30 min post-application and transported to the lab for larval bioassays with each cup receiving 20 early 3^rd^ instar *Ae*. *albopictus* larvae. A separate laboratory control group was tested concurrently in three replicates in 250 ml dechlorinated water. Larval mortality was recorded after 24 h with moribund larvae recorded as dead. Larval food (Brewer’s yeast, 30 mg/L) was provided during the bioassay.

### Autodissemination field efficacy

**Fig 2:**
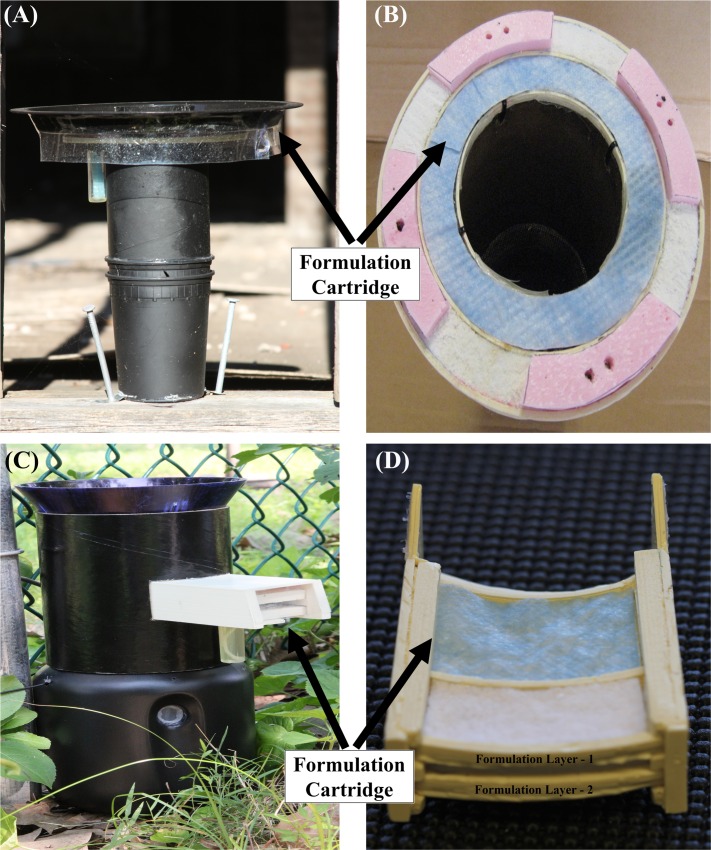
Autodissemination stations: (A) Model 2013 with top circular formulation cartridge. (B) A cartridge containing inner oil and outer powder formulation bands in 2013 model. (C) Model 2014 with side formulation cartridge. (D) A cartridge containing a dual layer of inner oil and outer powder formulation bands in 2014 model.

Autodissemination studies were conducted on the same Mercer and Essex Counties experimental plots as the sprayer experiments. Each 4000 m^2^ treatment plot was divided into four ca. 1000 m^2^ test blocks, with each block receiving one station in 2013 and two stations in the 2014 study. Dual phase autodissemination stations [14] were used in 2013, whereas the stations tested in 2014 were slightly modified. The major differences between 2013 and 2014 stations were the configuration of formulation cartridges. The autodissemination station in 2013 contained a circular dual band formulation cartridge (inner oil and outer powder bands) situated at the top of the transfer chamber (Fig 2A and 2B). To reduce insecticidal load per autodissemination station and economical cost, a dual layer formulations cartridge situated at the side wall of transfer chamber was used in 2014 (Fig 2C and 2D). Instead of single formulation ring in 2013, dual layer cartridge system in 2014 provided an opportunity for the station to perform even if one formulation layer fails under field conditions. Autodissemination stations were loaded with oil (20% a.i.) and powder (60% a.i.) pyriproxyfen formulation.

Open and cryptic cups (12 each) were placed in the treatment plot under foliage but away from direct sunlight. The control plot received five open sentinel cups. To increase the oviposition attractancy, stations and sentinel cups were filled with 1000 ml and 250 ml of oak infusion, respectively [15]. The day stations were deployed was considered “week 0”. Water samples were collected at weeks 2, 4 and 8 in 2013, whereas, an additional sample was collected at week 12 in 2014. At each collection event, infusion from the field ovicups was transferred to a new container after swirling, and the cups were refilled with 250 ml of infusion and returned to the field. Cups with less than 250 ml of infusion due to evaporation were replenished with tap water to the required volume before sampling. Collected samples were transported to the laboratory and tested for pyriproxyfen activity by larval bioassay.

Samples were filtered in the lab with a paper towel (Bounty® Procter and Gamble, USA) to remove organic matter and wild mosquito populations. From each sample, 200 ml of infusion was taken for bioassay to determine insecticidal activity and the remaining 50 ml was stored in an amber colored glass bottle at -20°C for subsequent residue analysis. Each bioassay container received 20 early 3^rd^ instar *Ae*. *albopictus* larvae. Pupal mortality was recorded as pyriproxyfen activity and larval mortality was excluded from analyses. Incomplete emergence or dead adult with attached exuviae were also considered as pupal mortality. For each experiment, an additional laboratory control was placed in three cups filled with dechlorinated water. Brewer’s yeast (30 mg/L) was provided as larval food twice a week. Cups were observed until either all individuals emerged as adults or died. Samples showing complete emergence inhibition or 100% pupal mortality were sent for residue analysis at Golden Pacific Laboratory, California, USA using LC-MS-MS analysis as described previously [14].

### Statistical analysis

Data were first tested for normal distribution using Shapiro-Wilk test. Data are presented as mean ± SE unless otherwise stated. Mann-Whitney Rank sum test was performed to compare eggs received by open and cryptic cups in tunnel experiment. Fisher Exact test was used to compare oviposition receiving and non-receiving, cryptic and open cups. Mann-Whitney Rank sum tests were performed for pairwise analysis to examine statistically significant differences for droplet density and median droplet size between open and cryptic cups. Efficacy of larvicide and pyriproxyfen autodissemination was expressed as percent larval and pupal mortality, respectively. In all bioassay experiments, control mortality was adjusted with open and cryptic cups mortality using Abbott’s formula [16]. Mann-Whitney tests were used to determine the significant difference in larval mortality between open and cryptic cups during larvicide application, and in pupal mortality during autodissemination experiments. Percent pyriproxyfen contamination was calculated using the following formula:

$$\%\;pyriproxyfen\;contamination=\frac{Number\;of\;containers\;showing\;pyriproxyfen\;activity}{Total\;number\;of\;containers}\times 100$$

## Results

### Oviposition preference

*Aedes albopictus* females showed a strong preference for oviposition in cryptic cups compared to open cups. Females deposited 83.8% (53.1±12.9 eggs/cup) of their eggs in cryptic cups, whereas open cups received only 16.2% (10.3±4.3 eggs/cup) (Mann-Whitney, T = 160, *p* = 0.002). Nearly 93% of cryptic cups received eggs, which was significantly higher than the open cups (40%) (Fisher Exact, *p* = 0.005).

### Droplet penetration

**Fig 3:**
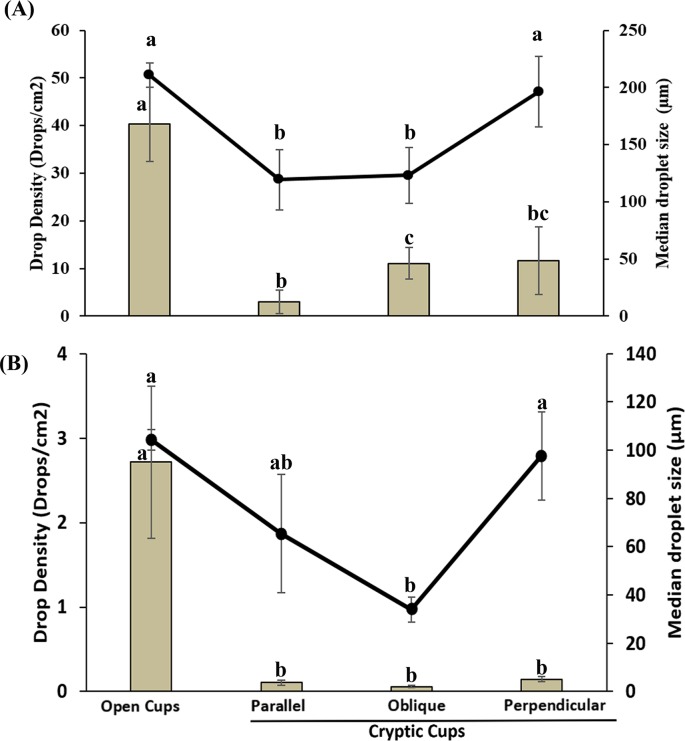
Droplet density (bar) and median size (line) in open and cryptic cups using backpack (A) and truck-mounted LV (B) sprayers. Letters above the bars and lines indicate significant differences (*p*<0.05). Data are shown as mean ± SE.

Backpack and truck mounted sprayers were highly effective at delivering spray droplets to open cups but ineffective at delivery to cryptic cups (Fig 3). With the backpack sprayer, open cups received the highest droplet density (40.3±7.9 droplets/cm^2^) followed by cryptic cups hidden inside pipes in perpendicular (11.7±7.1 droplets/cm^2^), oblique (11.1±3.3 droplets/cm^2^) and parallel (3.0±2.5 droplets/cm^2^) positions (Fig 3A). Irrespective of their position relative to spray direction, droplet density was significantly lower in cryptic than open cups (Mann-Whitney, parallel, T = 55, *p* = 0.001; oblique, T = 80, *p* = 0.019; perpendicular, T = 74, *p* = 0.008). Within cryptic cups, there was no significant difference between parallel and perpendicular cryptic cups and between oblique and perpendicular cups, however, droplet density in parallel cups was significantly lower than the oblique cups (Mann-Whitney, T = 58, *p* = 0.017). Median droplet diameter from the backpack sprayer was 174.82 μm (range, 51.37 to 390.66 μm) (Fig 3A). Droplets received by cryptic cups inside parallel and oblique pipes were smaller than the droplets received by open cups (Mann-Whitney, parallel, T = 68, *p* = 0.003; oblique, T = 74, *p* = 0.008). Droplet sizes received by open and cryptic cups in the perpendicular pipes did not differ significantly.

Similar droplet penetrations were observed from the truck mounted LV sprayer (Fig 3B). Open cups received the highest droplet density (2.72±0.9 droplets/cm^2^) followed by cryptic cups in perpendicular (0.15±0.03 droplets/cm^2^), parallel (0.11±0.03 droplets/cm^2^) and oblique (0.06±0.01 droplets/cm^2^) pipes. Irrespective of position, droplet density in cryptic cups was significantly lower than open cups (Mann-Whitney, parallel, T = 45, *p*≤0.001; perpendicular, T = 46, *p*≤0.001; oblique, T = 45, *p*≤0.001). There was no significant difference in droplet density within cryptic cups regardless of their position. Median droplet size of droplets produced by the LV sprayer was 95.0 μm diam (range, 12.67 to 180.75 μm) (Fig 3B). The size of droplets received by cryptic cups in the oblique pipe was significantly smaller than the open cups (Mann-Whitney, T = 45, *p*≤0.001).

### Sprayer field efficacy

The backpack application of *B*. *thuringiensis* larvicide produced 75.8±7.0% *Ae*. *albopictus* mortality in open ovicups, whereas cryptic cups showed virtually no measurable mortality at 0.3±0.3 (Mann-Whitney, T = 1867, *p*≤0.001). The truck-mounted treatment was similarly ineffective against cryptic cups (0.1±0.1% mortality) in comparison to open cups (19.4±6.1%) (Mann-Whitney, T = 1499, *p* = 0.001). Cups placed in the control plot did not show larval mortality in these trials.

### Autodissemination field efficacy

**Fig 4:**
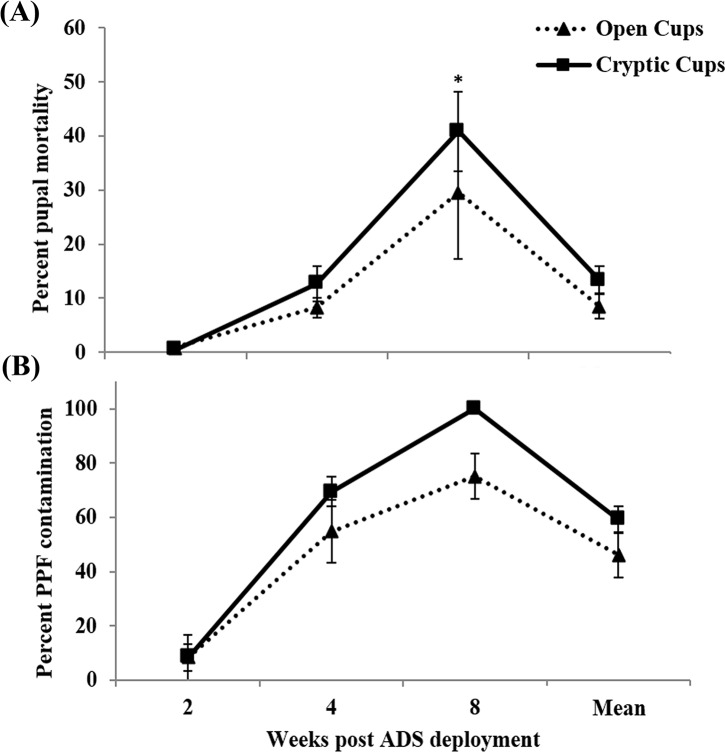
Weekly percent pupal mortality (A) and pyriproxyfen contamination (B) in open and cryptic cups during 2013 field experiment. Significant difference indicated by * (*p*<0.05). Data are shown as mean ± SE.

In the 2013 autodissemination experiment, mean percent pupal mortality in open cups was 8.4% (range, 0.9 to 29.6%) with highest pupal mortality was recorded in week 8 (29.6%) samples (Fig 4A). In cryptic cups, mean percent pupal mortality was 13.4% (range, 0.4 to 40.8%) with highest pupal mortality in week 8 samples (Fig 4A). No significant difference in pupal mortality was found between open and cryptic cups in weeks 2 and 4, although week 8 samples showed significantly greater mortality in cryptic than open cups (Mann-Whitney, T = 136, p = 0.03) (Fig 4A). Mean pyriproxyfen contamination in cryptic and open cups was 59.3±4.6% (range, 8.3 to 100%) and 46.0±8.2% (range, 8.3 to 75%) respectively, however, the difference was not significant (Fig 4B). Autodissemination was further confirmed by residue analysis from samples exhibiting high pupal mortality, with twice the concentration found in cryptic as compared to open cups (1.64 and 0.87 μg/L respectively).

**Fig 5:**
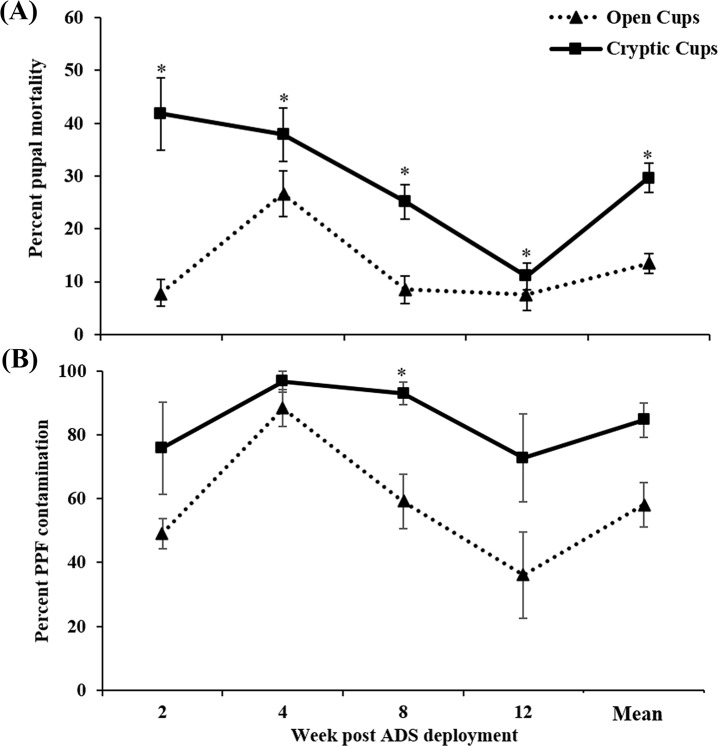
Weekly percent pupal mortality (A) and pyriproxyfen contamination (B) in open and cryptic cups during 2014 field experiment. Significant difference indicated by * (*p*<0.05). Data are shown as mean ± SE.

The number of autodissemination stations in treatment plots was increased in 2014 to increase the probability of pyriproxyfen transfer. Mean percent pupal in open cups was 13.4% (range, 7.5 to 26.6%), whereas in cryptic cups it was 29.7% (range, 11.0 to 41.8%) (Fig 5A). Pupal mortality in sentinel cryptic cups was consistently greater than open cups throughout the study (Fig 5A) (Mann-Whitney, week 2, T = 711, *p*≤0.001; week 4, T = 942, *p* = 0.025; week 8, T = 486, *p*≤0.001; week 12, T = 583, *p* = 0.04; overall, T = 15543, *p*≤0.001). Pyriproxyfen contamination ranged from 36.1 to 88.4% (mean, 58.2±6.9%) in open cups and 72.9 to 96.7% (mean, 84.6±5.3%) in cryptic cups (Fig 5B). Residue analysis confirmed the transfer of pyriproxyfen from contaminated mosquitoes into open and cryptic cups at 0.0046 and 0.0103 μg/L, respectively.

## Discussion

Asian tiger mosquitoes can use almost any small, water-holding container for larval development; however, this species showed a strong oviposition preference for cryptic over open cups in our field cage experiment suggesting a higher contribution of cryptic habitats in population buildup. The major difference between open and cryptic cups is their accessibility, open cups have a highly apparent and accessible top entrance, whereas cryptic cups are hidden within a pipe and only accessible from side entrances. Greater *Ae*. *albopictus* larval prevalence in corrugated extension tubes than open (i.e., exposed) containers in urban residential areas has been previously reported [17]. Higher larval prevalence was found in containers such as tires, water bottles, tin cans, gutter hoses, corrugated pipes, and water drainage pipes etc. [6,18–22] which displayed some degree of a cryptic nature.

If our hypothesis is correct that cryptic habitats hold much or most of the *Ae*. *albopictus* population, effective control strategies to curtail container mosquitoes in these habitats would be vital. Yet the droplet data demonstrated for the first time that cryptic cups are nearly impenetrable to the conventional backpack and truck mounted applications routinely used for larvicide applications [9,23]. Irrespective of their position relative to the spray application path, cryptic cups were difficult to reach by aqueous droplets. Similarly, cryptic cups were nearly invulnerable to our *Bti* applications as reflected in almost negligible larval mortality compared to open cups. The side entrance of cryptic cups makes them almost inaccessible to insecticidal droplets suggesting that the efficacy of conventional sprayers against container mosquitoes may be limited and their efficacy is overestimated when evaluated against open cups only as the majority of the population preferred cryptic habitats.

For effective management of the Asian tiger mosquito, an alternate vector management strategy is needed which can specifically target cryptic habitats. Autodissemination is emerging as a potential new strategy with a promise to deliver insecticides to both exposed and hidden habitats. Autodissemination exploits the behavior of the insect as a vehicle to deliver toxicants to the pest habitats. Consider the goat moth, *Coccus coccus*, which also lives in cryptic, hidden habitats. Pre-infecting adults with entomopathogenic nematodes and then releasing them to locate and deliver the parasites to these hard-to-reach target sites resulted in 86% larval mortality, whereas conventional methods produced only 4% larval mortality [24]. Similarly, the ability of autodissemination to transfer lethal concentrations of insecticides has been successfully demonstrated in the laboratory and the field for *Ae*. *albopictus* and other mosquito vectors [12,25–29]. Previous studies, however, have used only open cups to assess autodissemination efficacy and did not examine efficacy in cryptic habitats. We have tested and demonstrated that autodissemination can deliver toxic concentrations of insect growth regulator to both open and cryptic cups.

We have developed multiple designs for autodissemination stations that exploit the oviposition behavior of gravid *Ae*. *albopictus* to deliver pyriproxyfen to larval habitats [12,14,30]. Attracted to the station by an oak infusion, the mosquito enters the station but is unable to reach the water and oviposit. In the current design, when they exit they are forced to walk across an oil and powder fabric bands impregnated with pyriproxyfen. In addition to its insecticidal role, the oil formulation serves additional roles as a sticker to enhance attachment of the powder formulation, and to facilitate quick release into the larval habitat during oviposition.

In two years of field studies, we have shown here that autodissemination stations are useful against both open and cryptic cups. We observed higher pupal mortality and pyriproxyfen contamination in cryptic than open cups, however, this difference was not significant in 2013. Although pyriproxyfen reaches both cryptic and open cups in 2013, overall field efficacy was at best modest that year. Our 2013 plots were located in a suburban area with unmistakably low mosquito populations; because autodissemination relies entirely on wild adult mosquitoes to transfer the insecticide, the outcome from low populations cause low mortality. Greater autodissemination efficacy was obtained in the 2014 field study because stations were modified to perform for a longer period, the number of stations per block was doubled, and the trial was conducted in an inner-city area where adult populations were chronically high. Pyriproxyfen autodissemination in cryptic cups was consistently higher than open cups during the entire study period. Mortality in 2014 was comparable to previous autodissemination field experiments [30], though the pupal mortality in cryptic cups was relatively low to reach the desired level of effective control recommended by vector control authorities. Efficacy may have been underestimated because field samples were filtered to remove organic debris which likely reduced pyriproxyfen given this chemical’s propensity for adsorption to different substrates [31].

Pyriproxyfen dissemination was further confirmed by residue analysis of the sentinel cups, while previous autodissemination field studies have tended to rely on larval bioassay to show pyriproxyfen presence and activity but lacked direct evidence of transfer to sentinel cups [25,27,28]. Although, open and cryptic cups have shown the presence of pyriproxyfen, we found a surprisingly large difference in pyriproxyfen concentration between the 2013 and 2014 samples. One possible explanation for such a huge difference may be variation in the samples. During 2013, we analyzed week 8 samples for residue analysis. Whereas in 2014, week 4 samples were analyzed instead due to low pupal mortality in week 8 and week 12 samples. There is a possibility that pyriproxyfen may have accumulated in field sentinel cups over time in 2013, which eventually resulted in higher concentrations. However, this requires further investigation to establish the relationship of various factors *i*.*e*. age of water sample, organic components, temperature, and mosquito density that can affect pyriproxyfen residual concentrations.

Autodissemination system is a low maintenance and easy to operate which can cover an entire mosquito season once deployed. Pyriproxyfen is a highly effective juvenile hormone analog, besides its pupicidal action, pyriproxyfen sterilizes adult females and decreases spermatogenesis in males [32], shows ovicidal activity [13], and can terminate egg diapause prematurely [33]. The impact of pyriproxyfen on non-targets is minimal as dissemination is tightly targeted to small, scattered, container habitats. Additionally, the amount carried by females is minuscule which further reduces insecticide load in the environment [14].

## Conclusions

Conventional methods of evaluating larvicidal sprays through the placement of open sentinel cups may overestimate efficacy if much of the container larval population are found in their preferred cryptic habitats. Greater consideration to cryptic habitats may improve mosquito control programs and likely to have limited efficacy without specifically targeting cryptic habitats. Our results provide evidence that female-driven autodissemination can be a viable control strategy to suppress *Ae*. *albopictus* mosquitos in hidden habitats which are otherwise difficult to reach by conventional methods. Although autodissemination is not likely an area-wide approach, it shows great prospects for treating ‘hot spots,’ particularly where larval habitats are difficult to locate and where mosquito control agencies lack access due to restricted entry. Autodissemination stations provide an opportunity to enhance larval site treatment coverage for mosquito control and may be a powerful tool to enhance integrated vector management and target-based insecticide applications.
